# Recommendations for Implementing Lung Cancer Screening with Low-Dose Computed Tomography in Europe

**DOI:** 10.3390/cancers12061672

**Published:** 2020-06-24

**Authors:** Giulia Veronesi, David R. Baldwin, Claudia I. Henschke, Simone Ghislandi, Sergio Iavicoli, Matthijs Oudkerk, Harry J. De Koning, Joseph Shemesh, John K. Field, Javier J. Zulueta, Denis Horgan, Lucia Fiestas Navarrete, Maurizio Valentino Infante, Pierluigi Novellis, Rachael L. Murray, Nir Peled, Cristiano Rampinelli, Gaetano Rocco, Witold Rzyman, Giorgio Vittorio Scagliotti, Martin C. Tammemagi, Luca Bertolaccini, Natthaya Triphuridet, Rowena Yip, Alexia Rossi, Suresh Senan, Giuseppe Ferrante, Kate Brain, Carlijn van der Aalst, Lorenzo Bonomo, Dario Consonni, Jan P. Van Meerbeeck, Patrick Maisonneuve, Silvia Novello, Anand Devaraj, Zaigham Saghir, Giuseppe Pelosi

**Affiliations:** 1 Faculty of Medicine and Surgery—Vita-Salute San Raffaele University, 20132 Milan, Italy; veronesi.giulia@hsr.it; 2 Division of Thoracic Surgery, IRCCS San Raffaele Scientific Institute, 20132 Milan, Italy; pierluigi.novellis84@gmail.com; 3 Department of Respiratory Medicine, David Evans Research Centre, Nottingham University Hospitals NHS Trust, Nottingham NG5 1PB, UK; David.Baldwin@nottingham.ac.uk; 4 Department of Radiology, Icahn School of Medicine at Mount Sinai, New York, NY 10029, USA; claudia.henschke@mountsinai.org (C.I.H.); ntriphuridet@gmail.com (N.T.); rowena.yip@mountsinai.org (R.Y.); 5 Department of Social and Political Sciences, Bocconi University, 20136 Milan, Italy; simone.ghislandi@unibocconi.it (S.G.); lucia.fiestas@phd.unibocconi.it (L.F.N.); 6 Department of Occupational and Environmental Medicine, Epidemiology and Hygiene, Italian Workers’ Compensation Authority (INAIL), 00078 Rome, Italy; s.iavicoli@inail.it; 7 Center for Medical Imaging, University Medical Center Groningen, University of Groningen, 9712 CP Groningen, The Netherlands; m.oudkerk@umcg.nl; 8 Department of Public Health, Erasmus MC—University Medical Centre Rotterdam, 3015 GD Rotterdam, The Netherlands; h.dekoning@erasmusmc.nl (H.J.D.K.); c.vanderaalst@erasmusmc.nl (C.v.d.A.); 9 The Grace Ballas Cardiac Research Unit, Sheba Medical Center, Affiliated with the Sackler Faculty of Medicine, Tel-Aviv University, 52621 Tel Aviv-Yafo, Israel; joseph.shemesh@sheba.health.gov.il; 10 Roy Castle Lung Cancer Research Programme, Department of Molecular and Clinical Cancer Medicine, The University of Liverpool, Liverpool L69 3BX, UK; J.K.Field@liverpool.ac.uk; 11 Department of Pulmonology, Clinica Universidad de Navarra, 31008 Pamplona, Spain; jzulueta@unav.es; 12 Visiongate Inc., Phoenix, AZ 85044, USA; 13 European Alliance for Personalised Medicine (EAPM), Avenue de l’Armée Legerlaan 10, 1040 Brussels, Belgium; denis.horgan@euapm.eu; 14 Thoracic Surgery Department, University and Hospital Trust—Ospedale Borgo Trento, 37126 Verona, Italy; maurizio.infante@aovr.veneto.it; 15 Division of Epidemiology and Public Health, UK Centre for Tobacco and Alcohol Studies, Clinical Sciences Building, City Hospital, University of Nottingham, Nottingham NG5 1PB, UK; rachael.murray@nottingham.ac.uk; 16 The Legacy Heritage Oncology Center & Dr. Larry Norton Institute, Soroka Medical Center & Ben-Gurion University, 84101 Beer-Sheva, Israel; peled.nir@gmail.com; 17 Department of Medical Imaging and Radiation Sciences, IEO European Institute of Oncology IRCCS, 20141 Milan, Italy; cristiano.rampinelli@ieo.it; 18 Department of Surgery, Memorial Sloan Kettering Cancer Center, New York, NY 10065, USA; roccog@mskcc.org; 19 Department of Thoracic Surgery, Medical University of Gdańsk, 80-210 Gdańsk, Poland; wrzyman@gumed.edu.pl; 20 Department of Oncology, University of Torino, 10124 Torino, Italy; giorgio.scagliotti@unito.it (G.V.S.); silvia.novello@unito.it (S.N.); 21 Department of Health Sciences, Brock University, 1812 Sir Isaac Brock Way, St Catharines, ON L2S 3A1, Canada; martin.tammemagi@brocku.ca; 22 Division of Thoracic Surgery, IEO European Institute of Oncology IRCCS, 20141 Milan, Italy; luca.bertolaccini@gmail.com; 23 Faculty of Medicine and Public Health, Chulabhorn Royal Academy, HRH Princess Chulabhorn College of Medical Science, Bangkok 10210, Thailand; 24 Department of Biomedical Sciences, Humanitas University, 20090 Pieve Emanuele (MI), Italy; alexia.rossi03@gmail.com; 25 Department of Radiation Oncology, Amsterdam University Medical Centers, VU location, De Boelelaan 1117, Postbox 7057, 1007 MB Amsterdam, The Netherlands; s.senan@vumc.nl; 26 Department of Cardiovascular Medicine, Humanitas Clinical and Research Center IRCCS, 20089 Rozzano (MI), Italy; giuseppe.ferrante@humanitas.it; 27 Division of Population Medicine, School of Medicine, Cardiff University, Cardiff CF14 4YS, UK; brainke@cardiff.ac.uk; 28 Department of Bioimaging and Radiological Sciences, Catholic University, 00168 Rome, Italy; lorenzo.bonomo.md@gmail.com; 29 Epidemiology Unit, Fondazione IRCCS Ca’ Granda Ospedale Maggiore Policlinico, 20122 Milan, Italy; dario.consonni@unimi.it; 30 Thoracic Oncology, Antwerp University Hospital and Ghent University, 2650 Edegem, Belgium; jan.vanmeerbeeck@uza.be; 31 Division of Epidemiology and Biostatistics, IEO European Institute of Oncology IRCCS, 20141 Milan, Italy; patrick.maisonneuve@ieo.it; 32 Department of Radiology, Royal Brompton Hospital, London SW3 6NP, UK; a.devaraj@rbht.nhs.uk; 33 Department of Respiratory Medicine, Herlev-Gentofte University Hospital, 2900 Hellerup, Denmark; zaigham.saghir@gmail.com; 34 Department of Oncology and Hemato-Oncology, University of Milan, 20122 Milan, Italy; 35 Inter-Hospital Pathology Division, IRCCS MultiMedica, 20138 Milan, Italy

**Keywords:** consensus, statement, screening, lung cancer, mortality, reduction, low dose, computed tomography, implementation

## Abstract

Lung cancer screening (LCS) with low-dose computed tomography (LDCT) was demonstrated in the National Lung Screening Trial (NLST) to reduce mortality from the disease. European mortality data has recently become available from the Nelson randomised controlled trial, which confirmed lung cancer mortality reductions by 26% in men and 39–61% in women. Recent studies in Europe and the USA also showed positive results in screening workers exposed to asbestos. All European experts attending the “Initiative for European Lung Screening (IELS)”—a large international group of physicians and other experts concerned with lung cancer—agreed that LDCT-LCS should be implemented in Europe. However, the economic impact of LDCT-LCS and guidelines for its effective and safe implementation still need to be formulated. To this purpose, the IELS was asked to prepare recommendations to implement LCS and examine outstanding issues. A subgroup carried out a comprehensive literature review on LDCT-LCS and presented findings at a meeting held in Milan in November 2018. The present recommendations reflect that consensus was reached.

## 1. Introduction

Lung cancer is responsible for ~270,000 deaths annually in Europe, more than for any other cancer [1]. Despite long-standing interest in the European medical community for lung cancer screening (LCS) with low-dose computed tomography (LDCT) for reducing lung cancer mortality, supportive European data has only recently become available from a European Randomised Controlled Trial (NELSON). Use of LDCT-LCS in NELSON was associated with lung cancer mortality reductions of 26% in men and 39–61% in women [2]. These results have convinced experts and many politicians to advocate for LDCT-LCS implementation in Europe. However, the economic impact of LDCT-LCS still needs to be assessed, and guidelines for an effective and safe screening need to be formulated.

The Initiative for European Lung Cancer Screening, which comprises a large group of physicians and experts concerned with lung cancer, was convened to prepare recommendations on how LCS should be implemented in Europe and examine outstanding issues. A sub-group carried out a systematic review of the literature on LDCT-LCS and presented findings at a meeting held in Milan in November 2018. The present recommendations arose from that consensus was reached.

## 2. Eligibly Criteria for LDCT-LCS

### 2.1. Selection of High-Risk Individuals for LDCT-LCS

Screening is more effective in high risk individuals for lung cancer, but the selection of the population at risk amenable of screening (the common denominator to all evaluations) is of the utmost importance [3,4]. Current recommendations for high-risk individuals are based on either (a) criteria (mainly age and smoking history) originally used by the National Lung Screening Trial (NLST) [5], or derivative criteria, such as those introduced by the United States Preventive Services Task Force (USPSTF) [6,7] and the Centre for Medicare and Medicaid Services [8]; or (b) risk thresholds estimated by validated lung-cancer risk-prediction models.

Nearly 22 lung-cancer risk-prediction models have been published since 2003 [3,9,10,11,12,13,14,15,16,17,18,19,20,21,22,23,24,25,26,27], but many of them are considered unsuitable as they were validated in restricted or non-European populations or have only modest predictive power. Comparative studies [11,28,29] suggested that the models of Bach et al. [12], the Prostate, Lung, Colorectal, and Ovarian (PLCO) Cancer Screening Trial (PLCO_M2012_) [9], and the Lung Cancer Risk Assessment Tool perform better than others. PLCO_M2012_ has been validated in several countries [30,31]. The UK Lung Screen (UKLS) trial took advantage of the Liverpool Lung Project (LLP_v2_) risk model (5% risk over 5 years) to select high risk patients, identifying 2.1% lung cancers at baseline, which was higher than observed in either NLST or NELSON trial [32]. Recently, the NHS England Lung Health Projects [33,34] in the UK have used both the PLCO_M2012_ and the LLP_v2_ [35,36,37]. Probability thresholds adopted for screening eligibility with PLCO_M2012_ are ≥1.3% [38], ≥1.5% [3], and ≥2.0% [39,40].

When compared with criteria used in the NLST, risk models are likely to select older persons with a long smoking history and more comorbidities, who are also more likely to die from competing causes [41]. Nevertheless, as compared with NLST-like criteria, the best risk-prediction models have greater sensitivity and positive predictive value and lower number-needed-to-screen to avert one lung cancer death [3,9,10,11], although the observed superiority is more modest when considered gains of quality-adjusted life years (QALYS) [42].

Risk thresholds need to be periodically reassessed as the distribution of risk factors in a population may change over time and the utility of models may depend on either data availability or population traits. Moreover, the European Position Statement on Lung Cancer Screening [1] and the 2017 EU Policy Document on Lung Cancer Screening [43] emphasised that models should be able to identify individuals with sufficiently high risk to develop lung cancer in order to beneficially impact on the cost-effectiveness of LDCT-LCS. For example, LDCT-LCS is likely to be cost-ineffective in most cases of never-smokers [44], despite that lung cancer continues to remain one of the ten most frequent cancers in this demography group too. For the latter, LDCT-LCS might even be unethical, as harm could outweigh benefits [1].

In addition, clinicians are expected to play an important role in excluding from screening all individuals who are so frail or with reduced life expectancy that are unlikely to fruitfully benefit from curative intent treatments. The latter judgment is actually subjective and the keystone is to mimic the entry criteria of clinical trials. Models predicting competing causes of death and suggesting exclusion from screening or curative intent treatments are being developed: although they may prove useful to identify and manage (not over-treating) unlikely-to-benefit patients, they could lead to length time bias (overdiagnosis) [45,46,47].

Finally, a risk model incorporating lung nodule features and emphysema at baseline CT scan would allow recalculation of lung-cancer risk in screened persons [19,48,49]. The model is currently undergoing external validation and could help define optimal screening interval in a tailored way, thus significantly reducing overall costs and radiation exposure. Furthermore, there are machine-learning algorithms that could hopefully be part of artificial intelligence applications to predict the risk of malignancy in individual nodules sparing useless surgery.

In conclusion, identifying a lung-cancer risk model will help select high-risk individuals for LDCT-LCS in Europe is essential for the future of secondary prevention tools. Two candidate models—PLCO_M2012_ and LLP_v2_—may be suitable in this task. The former has been externally validated in North American datasets. The risk threshold should be either >1.5, >2 or more, over 6 years for PLCO_M2012_ and 2.5% or more over 5 years for LLP_V2_, depending on the national budget available. This recommendation is likely to change in the near future as prediction tools will become more extensively validated in Europe.

### 2.2. Inclusion of Asbestos-Exposed Individuals in LDCT-LCS

Asbestos is a major occupational and environmental carcinogen causing several cancers, particularly mesothelioma and lung cancer [50]. It was estimated in 2014 that 5–7% of newly diagnosed lung cancers were due to asbestos exposure [51]. The long latency of asbestos-related cancer development and the increasing life expectancy, surveillance and screening of asbestos-exposed persons may be instrumental to increase the proportion of individuals being diagnosed with early stage disease [28,50,51]. The 2015 Helsinki Consensus Report [52] had in fact recommended LDCT-LCS in the following workers: (a) those with any asbestos exposure and a smoking history according to NLST criteria (smokers and former smokers, aged 55–74, with pack-year ≥30); or (b) those with asbestos exposure regardless of smoking history but who have an estimated risk equal to that of NLST [5] study population. However, there is only limited evidence to support the use of LDCT-LCS in asbestos-exposed persons and other exposed workers [53,54]. Furthermore, most studies investigating LDCT-LCS in asbestos-exposed persons did not perform risk estimation to identify high-risk individuals for asbestos exposure [51]. Some lung-cancer risk-prediction models included asbestos exposure as a risk factor [14], but no validated model has comprised a detailed evaluation of asbestos variables in never-smokers. Such models are necessary to make LDCT-LCS in asbestos-exposed persons cost-effective and useful [51,52,55]. In the meantime, the criteria proposed by the 2015 Helsinki Consensus Report [52] for LDCT-LCS in asbestos-exposed persons may be adopted.

Unfortunately, identifying individuals exposed to asbestos is often a challenging task. In theory, asbestos-exposed persons might be monitored through databases generated by asbestos-using workplaces as per EU Directive 2009/148/EC. However, such data are often difficult to get. Use of validated questionnaires and checklists administered by trained interviewers are the most reliable means to identify persons with a work significant history of asbestos exposure [52,53,54,55,56], particularly when corroborated with data coming from the literature or databases on asbestos fibres content per air volume unit in workplaces [57,58]. Nevertheless, as people change jobs, it can be challenging to reconstruct occupational history and make precise estimations of asbestos exposure. Therefore, it is advisable cross-checking information upon questionnaires against data originated by trade unions, workers’ compensation and employment records, individual charts with diagnosed pleural plaques at chest X-ray and social security databases [56].

### 2.3. The LCS Recruitment Challenge in Europe

One of the major unresolved challenges to ensure optimal implementation of LDCT-LCS is participation, which is likely to result in lower accrual than other screening programs [39,59]. In particular, the participation rate of more-deprived socioeconomic status (SES) individual groups was low enough to be of concern for the successful implementation of LCS programs [39,59]. This is due to the over-representation of high-risk individuals in the more-deprived SES groups, despite that over 40% of detected lung cancers were in the most-deprived quintile people. This strong association with lower SES indicates that more-deprived groups will have to be included in future screening programs to reduce the existing inequalities in lung-cancer mortality. Although it might be difficult to recruit (former) smokers for intervention arms, benefits for the compliers may be substantial and even more than expected, since they might also benefit from other early evidence-based interventions, given the additional risk for other diseases.

This represents the most challenging balance to achieve between less costly and unsystematic approaches on the one hand (via open clinics, vans, advertisements), which likely will accrual lower risk individuals and systematic approaches on the other hand (via health registers, GP registers, questionnaires, and online surveys), which ensure better filtering of high-risk people but may be yet scarcely available and/or of unpredictable quality. Therefore, inviting individuals to LCS programs via existing and tested systems of patient accrual may be preferable. For example, in the Netherlands, which has population registries, the cancer-screening organisations could send a standard letter with just three questions on lung cancer concerns, residence characteristics (e.g., pollution) and smoking habit, along with a link to an online calculator [60]. However, even in the NELSON study, there was evidence that participants had slightly better self-reported health, with younger, more physically active, higher educated and more often former smokers comparable with eligible non-responders potentially more representative of the whole audience of lung cancer in the general population [60].

All these general approaches do not consider how different individuals will respond to different methods of invitation. Recruitment with a more “tailored approach” may be a solution, in which tailoring is used to (1) ensure all groups respond optimally; (2) allow risk assessment to be completed; (3) ensure that invitations optimise participation. This approach is especially important where population registries do not exist or are incomplete. The UK Lung Screen Uptake Trial (LSUT), which used a direct invitation strategy by primary care doctors in a deprived population, had a 53% participation rate [61]. The authors created a supportive and nonjudgmental service, acknowledging that the invited generation had been previously not as informed of the risks of smoking and, thus, avoided mentioning smoking, smoking cessation and risk where possible at the invitation stage. As a matter of fact, a potential risk of LCS, in the absence of adequate information and educational policy on smoking quitting, would be to create the false idea of an a priori protective effect of screening in subjects who continue to smoke undaunted.

### 2.4. Biomarkers for the Selection of Individuals for LDCT-LCS

Biomarkers may improve the effectiveness of screening by (a) refining the selection of persons for screening; (b) providing data indicating whether or not indeterminate screening-detected nodules are malignant; (c) predicting response to therapy and outcome. In theory, a single biomarker could be useful in all settings, but it is expected that risk-related markers will be more useful for accruing individuals to screening, whereas disease-related markers (e.g., tumour DNA) will be more useful in nodule management and predict outcome [62]. A risk marker for screening selection needs to be cheap, as it will be used on large numbers of people, whereas a disease marker has less stringent cost requirements, as it will be used on far fewer people. Irrespective of intended use, any biomarker should be able to detect lung cancer early in its preclinical phase, needs to be reliable, should be minimally invasive (e.g., applicable to biological fluids), be cost-effective and commercially available to disseminate its application.

Although numerous studies have evaluated biomarkers as indicators of lung cancer, no screening studies have included them as part of their protocol. Several reports have shown that panels of circulating tumour-related proteins can identify patients with lung cancer [63,64,65,66]. One study that detected auto-antibodies against a panel of six tumour-related antigens found that the panel identified 40% of primary lung cancers (only 33% of stage I), with a specificity of 90% vs. matched controls [66]. Another study detected a combined panel of circulating tumour DNA fragments and tumour-related proteins: 59% of newly diagnosed lung-cancer cases were uncovered with high specificity, but lower sensitivity for early-stage cancers [67]. More recently, a risk-prediction model based on smoking history and immunodetection of four protein markers (cancer antigen 125, carcinoembryonic antigen, cytokeratin-19 fragment, and precursor of surfactant protein B) in US cohorts was found to outperform a smoking-history-only model when applied to pre-diagnostic samples from two European cohorts [68]. At a fixed specificity of 0.83 based on the screening criteria of the United States Preventive Services Task Force (USPSTF), the model based on smoking-plus-biomarker had a sensitivity for screening eligibility of 0.63 vs. 0.42 for the smoking-based model alone.

Pre-diagnostic serum microRNA signatures have also been reported associated with lung cancer. Samples from 939 participants (69 with lung cancer; 870 disease-free) in the biological randomised Multicentre Italian Lung Detection Trial (BIO-MILD) were analysed using a quantitative PCR-based assay to derive a plasma microRNA signature cluster (MSC) [61]. The MSC had 87% sensitivity and 81% specificity across both arms and was able to predict the occurrence of lung cancer up to two years before its detection on CT scan [61]. Similarly, when validated in participants of the Continuous Observation of Smoking Subjects (COSMOS) study, the signature decreased to 77.8% sensitivity and 74.8% specificity [69,70].

Various other non-blood biomarkers have also been assessed for association with lung cancer, including volatile organic compounds from exhaled breath [71,72], condensate from exhaled breath [73], and bronchial epithelial cells in sputum after removal of non-bronchial cells [74]. Results are yet preliminary and need further investigation.

To conclude, biomarker assessment may improve eligibility selection for LDCT-LCS and management of screening-detected nodules. Panels of markers appear more promising than individual markers. However, most studies have assessed relatively small numbers of cases and several used samples from patients with a confirmed lung-cancer diagnosis. Larger studies on pre-diagnostic samples from longitudinally followed populations or screening cohorts are thus necessary to fully validate the performance of biomarker panels and justify their routine use in screening. Multinational studies on lung cancer-associated biomarkers are ongoing on mixed (smokers and non-smokers) and at-risk cohorts recruited to prospective screening trials. These studies are expected to provide more robust evidence on biomarker utility, but cost-effectiveness analysis will need to be nonetheless carried out for any new potential biomarker.

### 2.5. CT Protocols and Diagnostic Algorithms for Management of Nodules at Baseline and Repeat LDCT Scans

LCS is challenging at baseline, as findings have accumulated over a lifetime and may be of no clinical concern (length time bias). To minimise unnecessary harm and cost of work-up prior to the first annual repeat screening, work-up should be limited to participants with the highest suspicion of lung cancer while still aiming to identify small, early lung cancers. Cancers found in repeat rounds of screening are typically more aggressive and, thus, require work-up, so the timing should be different [75,76,77,78].

To assess published diagnostic protocols, a PROSPERO-registered systematic review of reports in English published before 9 November 2018, is currently underway. The primary outcome is the efficiency of the protocol (proportion of positive findings vs. lung cancer) by nodule type at baseline and repeat scans; secondary outcomes are the number of lung cancers detected per invasive work-ups, benign resection rate, and false-positive rate. Of the 9629 potential articles identified, only nine articles on eight separate studies qualified for inclusion: these included an international multi-institute study on 25,506 participants [79], a single institution study in Korea on 6406 participants [80], a single institution study in Ireland on 449 participants [81], a single institution study in Italy on 5201 participants [82], a 12-institute study in New York State on 6295 participants [83], a single institution study in Israel on 842 participants [84], a single institution study in Germany on 187 participants [85], and a single institution study in Taiwan on 3339 participants [86]. Three studies [79,83,84] used the I-ELCAP nodule-management protocol, whereas the others used separate study-specific management protocols. Preliminary results indicate that reporting on outcomes by nodule type and rounds of screening is limited, making it challenging to compare the efficiency of the different protocols.

In Europe, the four main protocols that have been used are the European consortium protocol based on the NELSON trial [2]; the I-ELCAP protocol [87], which has been used in Italy, Spain, and Switzerland; the American College of Radiology’s LungRADS; the British Thoracic Society Guideline (BTS) [88], which does not distinguish between incidental and screen-detected nodules [89]. A comparison of the baseline round of screenings of three of these protocols has been performed [79]. It determined the efficiency ratio (ER) of each recommendation by dividing the number of participants recommended for that work-up by the number of resulting lung-cancer diagnoses, a lower ER indicating that fewer participants undergo additional procedures for each diagnosis of lung cancer. For I-ELCAP, LungRADS Scenario 1 and Scenario 2, and the European consortium, ERs were, respectively, the following: for immediate work-up, 2.9, 8.6, 3.9, and 5.6; for delayed work-up, 36.1, 160.3, 57.8, and 111.9; overall, 13.9, 18.3, 18.3, and 31.9; for biopsies, 2.2, 8.1, 3.2, and 4.4. A low ER for biopsies is particularly important, as biopsies are invasive procedures, and unnecessary biopsies (i.e., of non-malignant nodules) should be minimised. All protocols use an initial LDCT in the screening round to determine who requires further short-term screening before the next annual repeat. The threshold values and the timing of the short-term follow-up are different. The important point of the comparison is that small differences in threshold values can lead to many unnecessary diagnostic work-ups and biopsies, as shown by the outcomes.

The BTS guideline [88] is the first to mandate the use of semi-automated volumetry in nodule management, and it is the recommended nodule-management method of the English Lung Health Check program.

All protocols should be reviewed and updated to incorporate advancing technology and knowledge, including the definition of positive results and the timing of further work-up. The work-up recommendations should be developed together with the relevant medical specialities, and it must be recognised that the LDCT findings are in asymptomatic participants and not in people seeking clinical care as prompted by symptoms.

For the future, machine learning may help in the detection and characterisation of nodules. Several publications have demonstrated the use of ML to characterise nodules from an image. Ardila et al. achieved 94.4% AUC performance on a large number of cases of the National Lung Cancer Screening Trial, and validation sets [90]. This creates an opportunity to optimise the screening process through IT assistance and case automation by enhancing advanced learning models in order to increase consistency and adoption of lung cancer screening worldwide.

### 2.6. Considerations on Volumetry and Doses

The NLST deemed ≥4-mm-sized nodules discovered at baseline screening to be suspicious for malignancy: this low threshold resulted in a high recall rate (27%) and a low positive predictive value (3.8%) [5]. A retrospective analysis of data from NLST and I-ELCAP suggested that the nodule-positivity threshold could be increased to 6 mm or even 8 mm (~300 mm^3^) to correct for the high false-positive rate in NLST [5].

The NELSON study assessed nodule volume and estimated volume-doubling times as an indicator of nodule growth rate and included a third category of undetermined nodules. Compared with NLST, this approach reduced positive test results at baseline to 2.6% of screened subjects and increased positive-predictive value to a satisfactory 36% [91].

Regarding the calculation of nodule volume, direct conversion of mean or maximum axial diameter to volume assuming sphericity leads to an overestimation of nodule volume as compared with semi-automated volumetry [92].

For any given volume, new nodules found at follow-up CT have a higher probability of being lung cancer than those detected at baseline [93,94]. Therefore, new (incident) nodules with volumes of 30–200 mm^3^ should be classified as indeterminate and require a repeat scan at 3 months to calculate the volume-doubling time; new nodules ≥200 mm^3^ should be referred to clinical work-up; in some occasions, a short observation period of one month and antibiotics can help reduce false positives for inflammatory disease at surgery or invasive procedures [82].

One of the concerns of CT screening is related to radiation exposure. There is no consensus on what level of radiation is considered ‘low-dose’. However, a low-dose lung cancer screening CT scan should be performed based on technical specifications ensuring that the quality of the screening and the radiation dose is in compliance with ACR-STR recommendations [95,96].

A recent study evaluating the cumulative radiation exposure and cancer risk from low-dose CT screening estimated a 0.05% additional risk of cancer after 10 years of screening and associated follow-up imaging [97]. However, different CT protocols and nodule management strategies in screening could lead to different levels of radiation exposure.

The assessment of cancer risk from radiation is based on the linear no-threshold model [98] and on data collected from occupational studies and from atomic bomb survivors. The risks are thus based on models generated from studies on people exposed to high levels of radiation; therefore, the linear no-threshold model stands as a precautionary recommendation that follows a conservative approach.

### 2.7. Work-Up and Treatment of Screening-Detected Nodules

To ensure successful LCS, it is essential to reduce to the minimum the number of invasive procedures for benign disease [99] and to avoid overtreatment of very early cancers or precancerous lesions. The best way to reduce surgery for benign lesions is to have an accurate preoperative/diagnostic algorithm, as this reduces the number of indeterminate nodules referred for surgery. In pre-specified cases, time should be allowed for watchful waiting to verify growth and calculation of volume-doubling time of the nodules and for repeated biopsies to substantiate malignancy. According to the National Comprehensive Cancer Network guidelines [100], a preoperative biopsy can be avoided when a strong clinical suspicion of Stage I or II lung cancer is present if the lesion is peripheral and if diagnosis can be easily obtained intraoperatively before resection [99].

The recommended threshold of surgical resection for benign disease should be below 10%. A percutaneous biopsy can assist in minimising benign resection rate and frozen section times [37]. Brock score can be used to triage patients between surveillance and further investigation [101].

For small and ground-glass nodules associated with early-stage cancers, sublobar resections—once reserved for functionally compromised patients—are being reconsidered. Limited resection, especially anatomical segmentectomy, may carry similar oncological outcomes as standard lobectomy, as demonstrated in retrospective studies [102,103,104], but the non-inferiority in oncological outcomes is still being assessed (trials JCOG 0802 and CALGB 140503) [105,106].

Minimally invasive techniques should be encouraged, post-operative 30-day mortality should be maintained lower than 1%, and major morbidity kept lower than 5%; the surgeons should be skilled in performing complex, minimally invasive anatomical sublobar resections (VATS or robotic segmentectomies) [107].

Recently published recommendations on the use of stereotactic body radiotherapy (SBRT) vs. surgery in early-stage non-small-cell lung cancer indicate a few main points when selecting patients for local radical treatment, [108] including: (a) for fit-for-surgery patients, SBRT is not contemplated outside the context of clinical trials, with the choice discussed within the MDT; (b) for high-risk patients, SBRT can be considered after adequate discussion within the MDT, provided patients are informed of decreased treatment-related risks and the unknown long-term outcomes; (c) SBRT should be carefully selected for central tumours, due to the increased risk of severe toxicity, one possible recommendation would be that the high-risk setting be defined by an FEV1 or a DLCO < 50%, or when there is a combination of risk factors, such as advanced age, impaired pulmonary function, pulmonary hypertension, or poor left-ventricle function. Nevertheless, the patient should have the final word after discussion with an expert thoracic surgeon. To aid the selection of candidates with borderline cardiopulmonary function, international guidelines and institutional adaptations of risk models are available [109,110]. If the MDT consensus and patient’s preference favours surgery, high-risk candidates should undergo sublobar or wedge resections through a minimally invasive approach [91,108,111]. For patients with second primary and multiple tumours, it is strongly recommended to discuss the patients within the MDT, and EBUS or mediastinoscopy be performed to rule out mediastinal involvement [112]. SBRT and/or surgery can be part of the same protocol for local aggressive treatment of oligometastatic disease [113].

### 2.8. Smoking Cessation and Other Initiatives within LDCT-LCS

#### Interventions to Stop Smoking

Patients undergoing LDCT-LCS may be particularly likely to consider stopping smoking, so may benefit from a smoking cessation (SC) initiative. However, data on the effectiveness of SC interventions in the context of screening are limited [114,115,116].

Five studies on smokers undergoing LCS without any formal SC program reported that 22% had stopped smoking two months after screening [117,118,119,120,121,122], 8% had stopped after six months [123], and ~14% were found, by biochemical assay, to be non-smokers a year after screening [124,125]. Ten studies reported outcomes of help given to all smokers undergoing screening [126,127,128,129,130,131,132,133,134,135]. Three studies investigated the effect of giving printed material only to encourage SC at randomisation. There were no significant differences in quit rates between the screened and control arms at two years [126,127], although in two studies screened-arm participants who underwent additional investigations were more likely to quit than controls or screened persons with a negative result [126,127]. One study reported that participation in LCS increased SC rates above that of the general population [128].

Studies reporting delivery of brief SC advice at LCS or during follow-up found quit rates of < 29% [129], but more typically 16–19.2% [130,131,132]. One large trial on the provision of minimal SC advice by a certified SC nurse to smokers attending LDCT-LCS reported no differences vs. control for up to five years’ follow-up [133,134]. A further study offering SC advice delivered by trained psychologists found a reduction in smoking prevalence at 2-year follow-up [135].

A small number of studies have compared SC interventions in LCS settings. No differences were found for standard written self-help materials vs. a list of internet SC resources [136], tailored vs. standard written SC information [137], or a brief SC counselling session on the day of screening vs. self-help printed materials and quit-line details [138]. One study testing the efficacy of six telephone-based services delivered by trained counsellors vs. self-help SC resources reported significantly higher biochemically verified 7-day point prevalent quit rates at 3-month follow-up vs. control (17.4% vs. 4.3%) [120]. One small study reported 57% abstinence for more than six months after cognitive-behaviour therapy therapy (CBT) and pharmacotherapy [122]; another study reported 20% biochemically validated continuous abstinence for one year in smokers who received four sessions of CBT-based telephone counselling by a psychologist and 12 weeks of pharmacotherapy with varenicline [119]. Finally, one trial tested the efficacy of e-cigarettes on smokers referred to LCS [118]: patients received monthly telephone SC counselling combined with either a nicotine-containing e-cigarette kit (intervention) or nicotine-free e-cigarettes (usual care). There was a significant reduction in daily cigarette consumption in the intervention group at one-month follow-up. Only one small trial investigated whether a tobacco-dependence treatment program was more effective when delivered before or after LDCT-LCS: it found higher quit rates when intervention was delivered before the scan [139].

Due to the small number of studies testing SC interventions at LCS have large variations in setting, participants, intervention, and outcome measure, they do not allow direct comparisons to be made. Thus, it is difficult to draw conclusions on the optimal intervention to administer. The SCALE collaboration in the US was established to support projects testing SC interventions delivered in LCS settings involving LDCT, and to build an evidence base for effective approaches [140].

Although data are lacking, the evidence does suggest that LCS in itself is a motivator for SC. However, more intensive SC interventions are needed to optimise quit rates within LCS programs, with smokers who do not receive the ‘all clear’ probably being more receptive.

### 2.9. Incidental Findings

Incidental findings are findings unrelated to lung cancer detection that are found because LDCT is a sensitive test for many different conditions. Correct management of incidental findings has the potential to increase benefit and cost effectiveness. However, incorrect management may lead to over-investigation and to treatment that may increase costs and harm patients.

#### 2.9.1. Incidental Findings of LDCT-LCS: Coronary Artery Calcification

Coronary artery calcification (CAC), extra-coronary cardiac and thoracic calcifications, aortic aneurysm, reduced bone density, and hepatic steatosis can all be recognised on LDCT scans, but they are reported inconsistently and there is no consensus on diagnostic criteria or clinical significances [141]. The coronary artery calcium score is the best single predictor of cardiovascular disease event and mortality, and equally, its absence is the best negative risk predictor [142,143,144,145]. Assessing CAC as part of LDCT-LCS can predict cardiovascular events, and consequently, reduce cardiovascular morbidity and mortality, and increase the cost-effectiveness of screening [146,147]. However, non-triggered LDCT CAC scoring may be associated with a higher false-negative rate.

Recently, American [148] and European [149] guidelines on cholesterol treatment confirmed the prognostic contribution of the CAC score as a risk category modifier of cardiovascular events and mortality (Class IIa). The Society of Cardiovascular Computed Tomography and the Society of Thoracic Radiology have jointly published guidelines for CAC scoring of noncontrast noncardiac chest CT scans, confirming the clinical use of combined lung screening and CAC score for cardiovascular re-categorisation. They recommended to report on CAC as a Class I indication, with reporting on thoracic aortic calcification as a Class IIb indication. For the scoring methodology, they recommended three classes: in Class I, CAC should be estimated as none, mild, moderate, or severe; in Class IIa, it is reasonable to perform an ordinal assessment of CAC on all noncontrast chest CT examinations; in Class IIb, it may be reasonable to perform Agatston CAC scoring on all noncontrast chest CT examinations.

It would be helpful to provide radiologists with information on how to diagnose, quantify, and report these incidental LDCT findings, and, in particular, to get expert consensus on routinely reporting CAC findings upon LCS. Indeed, CAC is easily detected and its extent can be quantified without extra radiation, with low additional effort, and cost. Most target subjects for LCS are also at high-risk for cardiovascular events and mortality, and there is robust evidence that CAC identifies high-risk patients and that treating them with statins improves outcomes. Thus, reporting CAC findings enhances the benefit of LCS by providing clinicians with a powerful, additive risk-stratification tool for the improvement of primary prevention of cardiovascular events.

Nevertheless, several questions need answering before a treatment based on CAC findings upon LDCT-LCS becomes established. Should all patients with incidentally diagnosed CAC be treated with preventive medications, such as aspirin or statins? Does treatment of risk factors based on CAC score reduce cardiovascular events? Should CAC findings be used to initiate medication in adults who would not otherwise qualify on the basis of current guidelines? To what extent should radiologists be involved in the clinical interpretation of CAC findings? Should the absence of CAC be used to reduce pharmacotherapy in individuals who may not need it?

#### 2.9.2. Incidental Findings of LDCT-LCS: Chronic Obstructive Pulmonary Disease

Patients selected for LDCT-LCS are more likely to have chronic obstructive pulmonary disease (COPD) because of common risk factors (age and tobacco smoking). COPD can be a marker of poor outcomes, based on severity and association with competing, smoking-related comorbidities; but because it is responsible for a third of deaths worldwide [150], LDCT is a sensitive test for emphysema, identifying previously undiagnosed, often asymptomatic, disease [151,152,153,154,155,156]. As expected, the prevalence of CT-detected emphysema is related to the quantity of tobacco smoked [153,157]. Therefore, the LCS-eligible population is likely to be COPD-enriched, with prevalence rates of 35–38% [151,154,156,157,158], almost four-fold higher than in the general population [159]. In the Lung Screen Uptake Trial—an observational cohort of 986 people at high risk of lung cancer invited for LDCT-LCS—spirometry consistent with COPD was observed in 57% of participants, whereas 10% with normal spirometry and no history of COPD had moderate/severe emphysema on CT [61].

Early detection of COPD may reduce mortality and morbidity, provided effective interventions can be deployed. However, detection of mild or asymptomatic COPD is presently considered not beneficial other than for supporting SC. Indeed, the USPSTF does not recommend identification of COPD in LDCT-LCS, citing insufficient evidence for impact on COPD-related endpoints [160]. Moreover, symptoms, exacerbation frequency, and comorbidities may be more important for prognosis than spirometry, hence the revised Global Strategy for the Diagnosis, Management, and Prevention of COPD (GOLD) criteria stressing symptom burden and exacerbation frequency over spirometry in guiding treatment [161]. Since about one-third of screened individuals have COPD [151,152,156], and studies have shown efficacy in reducing lung-cancer mortality, it is possible that LCS is effective in the reduction of mortality in COPD patients. Moreover, patients with COPD and/or emphysema have a two-to-three-fold greater risk of lung cancer [151,152,156]. Thus, identifying individuals with obstructive lung disease by spirometry may be useful in selecting individuals with a higher risk for lung cancer.

The presence of emphysema, airflow limitation, increasing COPD severity, and exacerbation frequency are associated with greater lung-cancer risk [156,162]. Indeed, in NLST participants with spirometry consistent with COPD, the incidence rate ratio for lung cancer vs. those without COPD was 2.15 [154]. Although much of this effect is explained by smoking [163], the addition of COPD history to multivariable risk-prediction models can improve predictive accuracy [151,156]. Furthermore, quantification of emphysema via LDCT may add to risk prediction by selection for subsequent LDCT-screening [157].

Radiological reporting of emphysema on LDCT predicts COPD in the LCS setting, with a sensitivity of 63% and specificity of 88% [158]. The recommendations reported by the Fleischner Society includes a visual, quantitative assessment of CT emphysema that may be too detailed for LDCT-LCS, given the minimal impact on outcomes [164].

What then is the potential benefit of LDCT-LCS detection of COPD? The evidence reviewed above suggests a beneficial effect provided there is existing COPD-derived morbidity. Most individuals in this category can be identified through questionnaires plus spirometry, and only a minority are identified solely through LDCT. The latter includes SC advice. Employing the latest GOLD recommendations can reduce COPD morbidity, and LCS may reduce mortality from lung cancer in these patients. Importantly, lung cancer is a leading cause of death in COPD patients.

Another potential benefit is increased SC in those diagnosed with COPD, especially as ongoing studies are evaluating whether making participants aware they suffer from emphysema, it favours SC. Moreover, the use of COPD in predictive models for lung cancer may also improve the effectiveness of screening by selecting those most likely to benefit. Finally, although COPD is a marker of other comorbidities that have effective interventions, it is unclear how COPD detection influences recognition of those comorbidities.

Enriching the screened population by using COPD as a risk factor may identify individuals at greater risk of death from competing causes. As comorbidities (coronary artery disease, heart failure, cardiac arrhythmias, hypertension, hypercholesterolemia, osteoporosis, diabetes) are frequent, they may benefit from treatment, but with a considerable reduction in quality-adjusted life years (QALY) [161,165]. Moreover, the ability to deliver effective treatments should be considered.

Thus, the benefit of CT-detected emphysema over spirometry assessment seems restricted to the identification of more patients possibly benefitting from targeted SC interventions and at future risk of clinically significant COPD. Nevertheless, detection of COPD and emphysema in LCS could be a teachable moment for SC, and an opportunity for lifestyle and pharmacological interventions to reduce COPD-related morbidity, particularly in those with symptoms and/or airflow limitation. Further prospective studies need to evaluate the impact on SC, COPD exacerbation, and hospitalisation in LCS participants.

### 2.10. Economic Policy for LCS and Educational Programs

#### Cost-Effectiveness of Mass LDCT Screening for Lung Cancer—A European Perspective

Following the findings of NLST, screening is now recommended in the US for high-risk individuals (as per USPSTF: age, 55–80 years; smoking history, at least 30 pack-years) [5,7]. Screening is now being implemented in the US, with private and public institutions covering costs [166,167]. In Europe, implementation has been slow, mainly because of fear of high costs to national health services and the general lack of cost assessments of LCS programs, except for in the UK [39].

Various cost-effectiveness analyses (CEAs) have been published. Early studies were from the USA [168,169,170] and Canada [171]. CEAs in those cases were found to vary substantially, from US$81,000 [168] to C$52,000 [171] per QALY gained. Notably, this variation did not prevent public authorities and insurance companies from supporting mass screening for high-risk persons. However, it is difficult to generalise these results to the European context. In particular, one important issue with the cost-effectiveness of screening programs is the evaluation of resource utilisation: although screening with LDCT is expensive, early cancer detection is usually cost-saving. Hence, the overall cost of a well-targeted screening program is an empirical issue that depends on the cost and regulatory structure of a national health system. Consumption calculations are, thus, very much country-specific, and so are CEA results.

In recent years, a series of CEAs for European countries have been published: in Germany, two studies found an incremental cost-effectiveness ratio (ICER) of €19,302 per life year (LY) gained (€30,241 per QALY) and €16,754–23,847 per LY [172,173]; in the UK, two studies found an ICER lower than £11,000 per QALY [39,174], whereas the most recent study found an ICER between £20,000 and £30,000 per QALY, but under extreme assumptions, such as very small cost advantage in treating Stages I and IV and no increase in average survival between the screening and no-screening scenarios [175]; in Poland, an ICER of €1353 per LY gained was found [176]. For Italy, our simulation-based model populated with real-world cost data from hospital records found an ICER of €3297 per QALY gained and €2944 per LY gained [177]: note that this is the most recent study in this sense, and it included immunotherapy for the treatment of Stages III and IV.

Although results are very heterogeneous, even within a given European country, overall the studies indicate favourable ICERs. Indeed, any value below £30,000 per QALY gained can be considered as evidence of the cost-effectiveness. Moreover, recent developments in the treatment of late-stage cases (with expensive immunotherapy) are likely to ameliorate the cost-effectiveness of screening even further in most countries.

## 3. Conclusions

The implementation of LCS with LDCT is urgently needed in Europe in order to diagnose lung cancer at an early stage and reduce mortality rates. European experts in this field agree that the knowledge developed during the last 20 years allows safe and effective programs to be implemented. Therefore, pilot LDCT programs should start in different countries. The UK and Poland have already established an institutional budget and started regional pilot LCS programs. Other countries are ready to start institutional health technology assessment for large-scale screening programs. The details of screening programs should be established in each country by an ad hoc commission.

For the sake of clarity and uniformity of screenings, our main recommendations are the following:

- a risk-prediction model (as LLP_v2,_ PLCO_M2012,_ or other) should be implemented to select high-risk populations;
- a key aspect for successful screening is the management of nodules, utilising volume and growth-rate data—more-stringent cut-offs are necessary for new nodules (detected at follow-up) than at baseline;
- LDCT protocol quality-control and assurance are essential;
- personalised screening-interval recommendations (annual vs. biennial) requires validation—currently, annual screening is recommended;
- national reference centres for quality assessment/control of the imaging process (equipment, facilities, personnel) should be linked across Europe;
- the management of incidental findings requires careful attention; stepwise implementation of CAC score and COPD should be embedded in LDCT-LCS;
- LDCT-LSC should be part of a defined national program to avoid low-quality opportunistic screening;
- effective evidence-based smoking cessation initiatives should be incorporated into LDCT-LSC programs.
- cost-effectiveness studies are required in Europe.

## Abbreviations

CAC	coronary artery calcification
CBT	cognitive-behaviour therapy
CEA	cost-effectiveness analysis
COPD	chronic obstructive pulmonary disease
ER	efficiency ratio
GOLD	Diagnosis, Management, and Prevention of COPD
ICER	incremental cost-effectiveness ratio
LCS	lung-cancer screening
LDCT	low-dose computed tomography
LY	life year
MSC	microRNA signature cluster
QALY	quality-adjusted life years
SBRT	stereotactic body radiotherapy
SC	smoking cessation
SES	socioeconomic status
COSMOS	Continuous Observation of Smoking Subjects study
I-ELCAP	Early Lung Cancer Action Program
LHLP	Liverpool Healthy Lung Program
LLP	Liverpool Lung Project
NELSON	European Randomised Controlled Trial (Dutch acronym)
NLST	National Lung Screening Trial
PLCO	Prostate, Lung, Colorectal, and Ovarian Cancer Screening Trial
UKLS	UK Lung Screen
USPSTF	United States Preventive Services Task Force
